# Oxidative Stress and Iron Addiction: A Comparative Study of 1321N1 Astrocytoma and T98G Glioblastoma Cells with Differential Expression of L-Cysteine-Metabolizing Enzymes

**DOI:** 10.3390/biom15101478

**Published:** 2025-10-20

**Authors:** Halina Jurkowska, Ewa Jasek-Gajda, Konrad Kaleta, Leszek Rydz, Dominika Szlęzak, Maria Wróbel

**Affiliations:** 1 Chair of Medical Biochemistry, Faculty of Medicine, Jagiellonian University Medical College, 7 Kopernika St., 31-034 Kraków, Poland; leszek.rydz@uj.edu.pl (L.R.); dominika.szlezak@uj.edu.pl (D.S.); mtk.wrobel@uj.edu.pl (M.W.); 2 Department of Histology, Faculty of Medicine, Jagiellonian University Medical College, 7 Kopernika St., 31-034 Kraków, Poland; ewa.jasek@uj.edu.pl; 3 Doctoral School of Medical and Health Sciences, Jagiellonian University Medical College, 16 Św. Łazarza St., 31-530 Kraków, Poland; konrad.kaleta@student.uj.edu.pl

**Keywords:** gliomas, cystathionine β-synthase, transferrin receptor 1, cysteine dioxygenase 1, sulfurtransferases, cystathionine-γ-lyase, sulfane sulfur, oxidative stress

## Abstract

Gliomas are central nervous system primary tumors that are distinguished by heterogeneity, broad-based infiltration, and metabolic reprogramming that sustains proliferation, invasion, and therapy refractoriness. Oxidative stress—a state of imbalance between the production of reactive oxygen species (ROS) and the antioxidant defense—and disturbed iron metabolism are central drivers of glioma biology. The aim of this study was to evaluate ROS production, sulfane sulfur levels, the expression of proteins with antioxidant properties, such as L-cysteine-metabolizing enzymes (cystathionine β-synthase, CBS; cysteine dioxygenase 1, CDO1; cystathionine γ-lyase, CTH; 3-mercaptopyruvate sulfurtransferase, MPST; thiosulfate sulfurtransferase, TST) and non-enzymatic proteins (p53; transferrin receptor 1, TfR1), in human brain cancer cells differing in malignancy: 1321N1 astrocytoma and T98G glioblastoma. Western blotting analysis demonstrated that the expression of CBS, CDO1, and TfR1 was significantly increased in T98G cells, while CTH, MPST, TST, and p53 were comparably expressed in both cell lines. Quantitative assays revealed that T98G cells harbored significantly higher sulfane sulfur levels and higher numbers of ROS-positive cells compared to 1321N1 cells. Our results suggest that glioblastoma but not astrocytoma cells adapt sulfur and iron metabolism to provide proliferation capacity against chronic oxidative stress. It seems that CBS as well as CDO1 may significantly increase the antioxidant potential of T98G cells. In summary, this study suggests a differing metabolic vulnerability identifiable only in high-grade glioma cells and provides a potential novel molecular target for therapy.

## 1. Introduction

Gliomas are a heterogeneous group of primary central nervous system (CNS) neoplasms, bounded by their glial origin but distinguished by extremely variable histology and molecular characteristics. The recent fifth WHO Classification of Tumors of the CNS (WHO CNS5, 2021) redefined the glioma nosology by introducing molecular diagnostics into tumor classification. Main adult-type diffuse glioma entities include Astrocytoma—IDH (isocitrate dehydrogenase)-mutant (WHO grades 2–4), Oligodendroglioma—IDH-mutant and 1p/19q-codeleted (grades 2–3), and Glioblastoma—IDH wildtype (grade 4) [1,2]. Gliomas account for ca. 24.5% of all primary CNS tumors and 80.9% of brain malignant tumors in adults [3]. In children, gliomas constitute ~45% of all CNS tumors, with diffuse midline glioma (31.1%), pilocytic astrocytoma (18.3%), and diffuse astrocytoma/anaplastic astrocytoma (5.3%) being the most encountered subtypes [3,4].

Glioblastoma, the most aggressive type, has an incidence rate of 3.23 per 100,000 adult individuals annually; diffuse astrocytomas as well as anaplastic astrocytomas occur at 0.46 and 0.42 per 100,000, respectively. In turn, oligodendrogliomas and anaplastic oligodendrogliomas occur at decreased rates (0.23 and 0.11 per 100,000, respectively) [3]. Gliomas are typically more prevalent in adults, with glioblastoma being diagnosed mostly in the sixth and seventh decades of life (median age: ~65 years), and diffuse astrocytomas and oligodendrogliomas diagnosed mostly in young adulthood (median ages: 46 and 43 years, respectively) [3]. Prognostically, gliomas exhibit significant variability. Five-year survival rates span from 94.7% in pilocytic astrocytoma to merely 6.8% in glioblastoma [3]. Standard-of-care treatment for glioblastoma includes maximal safe resection followed by focal radiotherapy (60 Gy) and concomitant temozolomide, yet median overall survival remains below 24 months, with a five-year survival rate under 10% [1,2]. Importantly, molecular stratification dramatically influences outcomes among low-grade gliomas. IDH-mutant, 1p/19q-codeleted tumors (oligodendrogliomas) may achieve median survival exceeding 14 years, whereas those with IDH wildtype low-grade tumors often demonstrate rapid progression and poor prognosis analogous to primary glioblastoma [2,3].

The pathogenesis of gliomas remains not fully explained. Most gliomas are sporadic but about 5% occur in the context of familial syndromes such as neurofibromatosis type 1 (NF1), tuberous sclerosis complex, Li–Fraumeni syndrome, Lynch syndrome, familial atypical multiple mole melanoma (FAMMM) syndrome, Ollier disease, and Maffucci syndrome [4,5]. Ionizing radiation is the only environmental risk factor universally recognized for the causation of gliomas. Children irradiated for benign or malignant diseases have a sevenfold elevation in secondary glioma risk, with maximum vulnerability in children irradiated before five years of age [3,5].

Glial lineage tumors are marked by extreme heterogeneity, diffuse brain infiltration, and a characteristic rewiring of redox metabolism that sustains proliferation, invasion, and therapy resistance [6,7,8]. An imbalance between the production of reactive oxygen species (ROS) and antioxidant defenses leading to oxidative stress is a defining feature of glioma biology: ROS are supposed to act as signaling second messengers that shape proliferation and angiogenesis, yet their chronic accumulation inflicts DNA, lipid, and protein damage, fostering genomic instability and mutational inactivation of tumor suppressors such as p53 [6,7,8,9,10]. The transcription factor NRF2 (Nuclear factor erythroid 2-related factor 2) orchestrates the cellular antioxidant defense controlling glutathione, thioredoxin, and iron/heme homeostasis, displaying a dual role in the brain: the preservation of neuronal–glial redox balance, yet, when persistently activated in glioblastoma, supporting glioma stem cell maintenance [6,8]. Iron metabolism integrates tightly with these redox circuits: iron drives ROS generation and DNA synthesis [11]. The transferrin receptor1 (TfR1/CD71) is the primary receptor for cellular iron uptake [12,13]. TfR1 consists of two disulfide-linked monomers; each monomer binds to one holo-Transferrin (Tf with iron), leading to the formation of the Tf-TfR complex, which enters the cell by endocytosis [13]. TfR1 is expressed on all cells, except mature erythrocytes and those terminally differentiated [12]. TfR1 is highly expressed in the brain capillary endothelium and neurons; its expression can be changed in tumors, including brain cancers [6,13,14].

**Figure 1:**
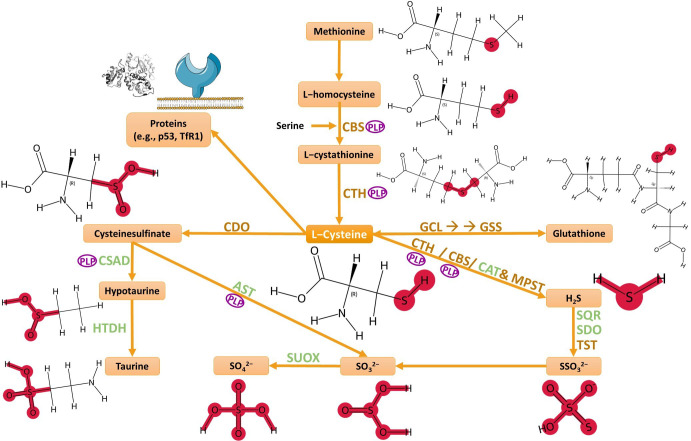
Biochemical transformations of L-cysteine. Abbreviations: CBS—cystathionine β-synthase; CTH—cystathionine γ-lyase; CAT—cysteine aminotransferase; MPST—3-mercaptopyruvate sulfurtransferase; CDO—cysteine dioxygenase; CSAD—cysteine sulfinic acid decarboxylase; AST—aspartate aminotransferase; HTDH—hypotaurine dehydrogenase; SUOX—sulfite oxidase; GCL—glutamate–cysteine ligase; GSS—glutathione synthetase; SQR—sulfide:quinone oxidoreductase; SDO—sulfur dioxygenase; TST—thiosulfate sulfurtransferase; PLP—pyridoxal phosphate. Image created with support from Servier Medical Art (https://smart.servier.com/), licensed under CC BY 4.0 (https://creativecommons.org/licenses/by/4.0/), accessed in 12 September 2025.

A central biochemical axis that links redox control in gliomas is the transsulfuration pathway and L-cysteine-metabolizing enzymes, such as cysteine dioxygenase 1 (CDO1), cystathionine β-synthase (CBS; EC 4.2.1.22), cystathionine γ-lyase (CTH; EC 4.4.1.1), 3-mercaptopyruvate sulfurtransferase (MPST; EC 2.8.1.2), and thiosulfate sulfurtransferase (TST; rhodanese; EC 2.8.1.1) (Figure 1) [15,16,17,18,19,20].

Both CBS and CDO1 are iron-dependent enzymes. CBS is made of four protein domains complexed around a heme group; as the iron oxidizes (Fe^3+^), CBS enters in its active conformation [21]. CDO1 is a non-heme iron (Fe^2+^)-dependent dioxygenase [22,23,24,25]. The structure of the active center of CDO1, which is the mononuclear iron center, is coordinated with the Nε2 atoms of three histidine ligands (His^86^, His^88^, and His^140^) and water molecules bound to the catalytic iron [25]. Stipanuk et al. reported [26] that cysteine dioxygenase is resolved into at least two bands upon SDS-PAGE electrophoresis—25 kDa and 23 kDa. The higher 25 kDa band is always observed, and the 23 kDa band is frequently observed, particularly in samples that contain a high concentration of these enzymes [26]. CDO1 converts cysteine into cysteine sulfinic acid (CSA), which is then catalyzed to hypotaurine and taurine or sulfate [26,27,28,29,30,31]. CDO1 is involved in many physiological processes, such as lipid metabolism, adipogenesis, redox homeostasis, fertility, osteoblastic differentiation, sulfide and bile acid metabolism, organismal growth and development [25].

CBS, CTH, MPST, and TST play important functions in sulfane sulfur-containing compounds formation and H_2_S metabolism [28,32,33,34,35,36,37,38,39,40,41,42,43]. Hydrogen sulfide and, critically, sulfane sulfur species—per- and polysulfidated small molecules and proteins—form a reactive sulfur signaling layer that intersects with bioenergetics, antioxidant defense, and cell death control in cancer, including gliomas [44]. Sulfane sulfur species act both as reservoirs for H_2_S and as autonomous effectors that modify protein cysteines by persulfidation, thereby tuning enzyme activities and redox sensors relevant to tumor metabolism [44]. More broadly across cancers, H_2_S exerts dose- and cellular condition-dependent effects on apoptosis: it can activate p53 and p38/MAPK signaling, increase ROS and sensitize glioblastoma lines to radiotherapy, or, conversely, bolster survival via NF-κB or PI3K/Akt pathways—implicating the quantitative and compartmental control of H_2_S/sulfane sulfur species as decisive for outcomes [45].

Recent attention has focused on sulfur metabolism, including pathways governing cysteine uptake, glutathione synthesis, and persulfide generation. These redox regulatory mechanisms are essential for glioma survival under oxidative stress, particularly in the context of hypoxia, high proliferation rates, and therapeutic resistance. Given the emerging evidence that molecularly defined glioma subtypes differ not only in genetics but also in metabolic programming, sulfur metabolic flux may represent a previously underappreciated axis of therapeutic vulnerability. Investigating the interplay between tumor genomics and sulfur metabolism may thus offer new avenues for subtype-specific, metabolically targeted interventions in the treatment of diffuse gliomas.

The aim of our study was to assess the correlation between ROS production, sulfane sulfur levels, and the expression of enzymatic proteins (CBS, CTH, TST, MPST, CDO1) and non-enzymatic proteins (TfR1, p53) involved in L-cysteine and iron metabolism, in human brain cancer cells differing in the degree of malignancy, such as 1321N1 (low-grade malignant astrocytoma) and T98G (grade IV glioblastoma multiforme).

By demonstration of the iron- and sulfur-dependent redox tumor control, this work seeks to provide a substantial basis for further research toward developing new therapies that target specific proteins involved in iron and sulfur metabolism in brain cancers. This goal is particularly relevant given the therapeutic constraints imposed by the blood–brain barrier, the proximity of tumors to eloquent brain regions, and the persistently poor five-year survival in this disease.

## 2. Materials and Methods

### 2.1. Cell Culture

The study was performed in two human glioma cell lines, 1321N1 and T98G, purchased from the European Collection of Authenticated Cell Cultures (ECACC, Salisbury, UK). The biological and genetic characteristics of both cell lines are shown in Table 1.

1321N1 and T98G cells were cultured in high-glucose (4.5 g/L) Dulbecco’s Modified Eagle Medium (DMEM) (Gibco-Thermo Fisher Scientific, Waltham, MA, USA) containing GlutaMAX and sodium pyruvate, to which 10% fetal bovine serum (FBS) (Biowest, Bradenton, FL, USA) and 1% penicillin/ streptomycin (100 Units/mL penicillin and 100 µg/mL streptomycin) were added. Cultures were maintained at 37 °C in a humidified atmosphere of 95% air and 5% CO_2_. Both cell lines were verified as Mycoplasma-negative.

### 2.2. Western Blot Analysis

1321N1 and T98G glioma cells were seeded at a density of 2.5 × 10^4^ per well in six-well plates. The cells were lysed in buffer (50 mM Tris–HCl, pH 7.5, 150 mM NaCl, 1 mM EDTA, 0.5% NP-40), supplemented with 1× Complete Protease Inhibitor Cocktail (Sigma-Aldrich Corp., St. Louis, MO, USA). Cell lysates were centrifuged at 20,000× *g* for 15 min at 4 °C. Protein concentrations were measured using the bicinchoninic acid (BCA) assay (Thermo Scientific/Pierce Biotechnology, Rockford, IL, USA). Samples (25 µg of protein) were subjected to electrophoresis on a 12% polyacrylamide gel (SDS-PAGE) and blotted onto 0.22 µm polyvinylidene difluoride (PVDF) membranes (Bio-Rad, Hercules, CA, USA). The membranes were blocked in 5% non-fat milk for 1 h, and then incubated overnight with the primary antibodies: anti-CBS (1:800; mouse monoclonal, #H00000875-M01, Abnova, Taiwan), anti-MPST (1:800; rabbit polyclonal, #GTX108274, GeneTex, Inc., Irvine, CA, USA), anti-TST (1:800; mouse monoclonal, #66018-1-Ig, Proteintech Group, Rosemont, IL, USA), anti-CTH (1:1000; mouse monoclonal, #60234-1-Ig, Proteintech Group, Rosemont, IL, USA), anti- CD71 (transferrin receptor 1; 1:500; rabbit polyclonal, #10084-2-AP, Proteintech Group, Rosemont, IL, USA), anti-CDO1 (1:500; rabbit polyclonal, #12589-1-AP, Proteintech Group, Rosemont, IL, USA), anti-p53 (1:800; mouse monoclonal, #05-224; Upstate & Chemicon, CA, USA), and anti-β-actin (1:1000; mouse monoclonal, #A1978, Sigma-Aldrich Corp., St. Louis, MO, USA). Goat anti-mouse and goat anti-rabbit alkaline-phosphatase-conjugated secondary antibodies (1:2000, Proteintech Group, Rosemont, IL, USA) were added for 1.5 h at room temperature. Immune complexes were visualized with nitro blue tetrazolium/5-bromo-4-chloro-3-indolyl phosphate (NBT-BCIP) stock solution (Roche Applied Science, Penzberg, Germany). Densitometric analysis was performed using the ChemiDoc™ MP Imaging System (Bio-Rad, Hercules, CA, USA). β-Actin was used as the internal loading control.

### 2.3. Determination of Sulfane Sulfur Level

The sulfane sulfur level was determined by using the method of Wood et al. [48], based on cold cyanolysis and colorimetric detection of ferric thiocyanate complex ions. The sulfane sulfur level was calculated as µmol of SCN^−^ produced per 1 mg of protein. The total protein content level was determined by using the method of Lowry et al. [49] with crystalline bovine serum albumin as a standard.

### 2.4. Flow Cytometry Analysis of ROS Production

The quantitative assessment of oxidative stress in 1321N1 and T98G glioma cells was carried out with the Muse Oxidative Stress Kit (Merck Millipore, Billerica, MA, USA) following the manufacturer’s protocol. This assay employs dihydroethidium (DHE), a cell-permeant probe that, upon oxidation by superoxide anions, is converted to a DNA-binding fluorophore, enabling discrimination of ROS-negative [ROS (−)] and ROS-positive [ROS (+)] sub-populations.

1321N1 and T98G cells were harvested (1 × 10^6^ cells mL^−1^), washed with PBS, and incubated in the dark at 37 °C for 30 min with the Muse Oxidative Stress Reagent working solution containing DHE. The percentage and absolute count of ROS (−) and ROS (+) cells were quantified with the Muse Cell Analyzer and Muse Analysis Software 1.3 (Merck Millipore, Billerica, MA, USA).

### 2.5. Statistical Analysis

All results are presented as the means ± standard deviation (SD). The normality of data distribution was assessed using the Shapiro–Wilk test. Statistical comparisons were performed using the Mann–Whitney U test. The results represent at least three independent experiments. Differences with *p*-values < 0.05 were considered statistically significant. Statistical analyses were conducted using GraphPad Prism 9.0 (GraphPad Software Inc., La Jolla, CA, USA).

## 3. Results

### 3.1. The Level of Sulfane Sulfur in 1321N1 and T98G Cells

**Figure 2:**
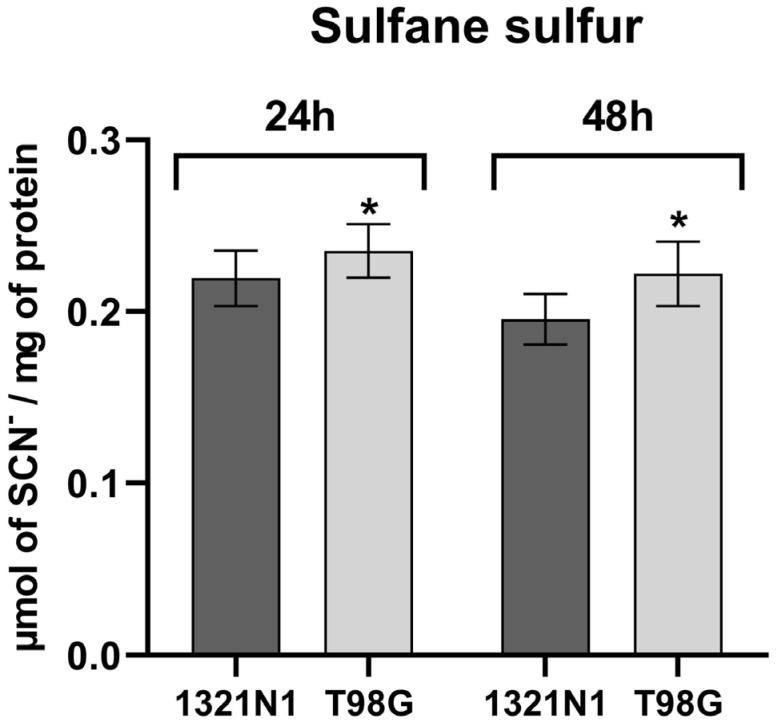
The level of sulfane sulfur in human 1321N1 and T98G cell lines. The sulfane sulfur level determined after 24 and 48 h of culture was calculated as micromoles of SCN^–^ per milligram of protein. The results are expressed as the mean ± SD of three or more independent experiments. An asterisk (*) indicates a statistically significant difference (*p* < 0.05) between cell lines at each time point, 24 and 48 h.

The intracellular level of sulfane sulfur was analyzed in both cell lines studied. We observed that the level of sulfane sulfur in T98G cells was higher than in 1321N1 cells after 24 h and 48 h of culture (Figure 2).

### 3.2. The Level of Proteins Involved in H_2_S and Iron Metabolism in 1321N1 and T98G Cells

**Figure 3:**
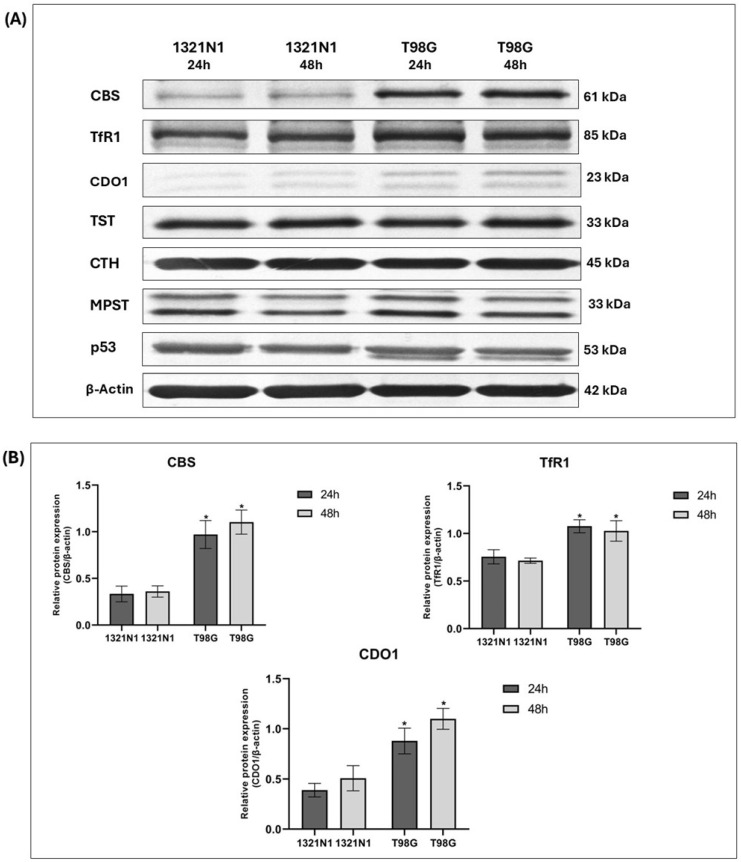

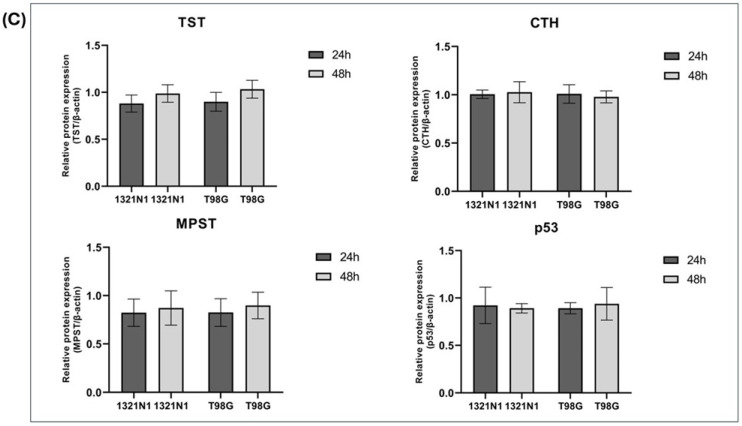
The expression of CBS, TfR1, CDO1, TST, CTH, MPST, and p53 at the protein level in human 1321N1 and T98G cancer cell lines after 24 and 48 h of culture (Western blot analysis). β-Actin was used as the loading control. (**A**) Data from a representative experiment are presented; the original images of Western blot results are shown in the Supplementary Materials. Quantification of the expression of (**B**) CBS, TfR1, CDO1, and (**C**) TST, CTH, MPST, p53 was performed by densitometric analysis of the blots and normalized to the internal loading control. The results are expressed as the mean ± SD of three or more independent experiments. An asterisk (*) indicates statistically significant differences between cell lines at each time point (*p* < 0.05).

In 1321N1 and T98G cells, the expression of CBS, TfR1, CDO1, TST, CTH, MPST, and p53 was studied at the protein level. The representative results of these studies are shown in Figure 3A.

The Western blot analysis showed that the expression of CBS, CDO1, and TfR1 is markedly higher in T98G cells compared to 1321N1 cells (after both 24 h and 48 h of culture) (Figure 3A,B). The expression of TST, CTH, MPST, and p53 was comparable in both studied cell lines (Figure 3A,C).

### 3.3. The Level of Reactive Oxygen Species in 1321N1 and T98G Cells

**Figure 4:**
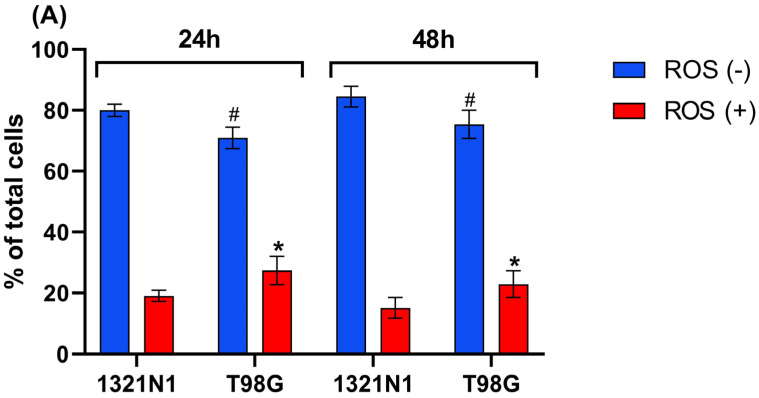

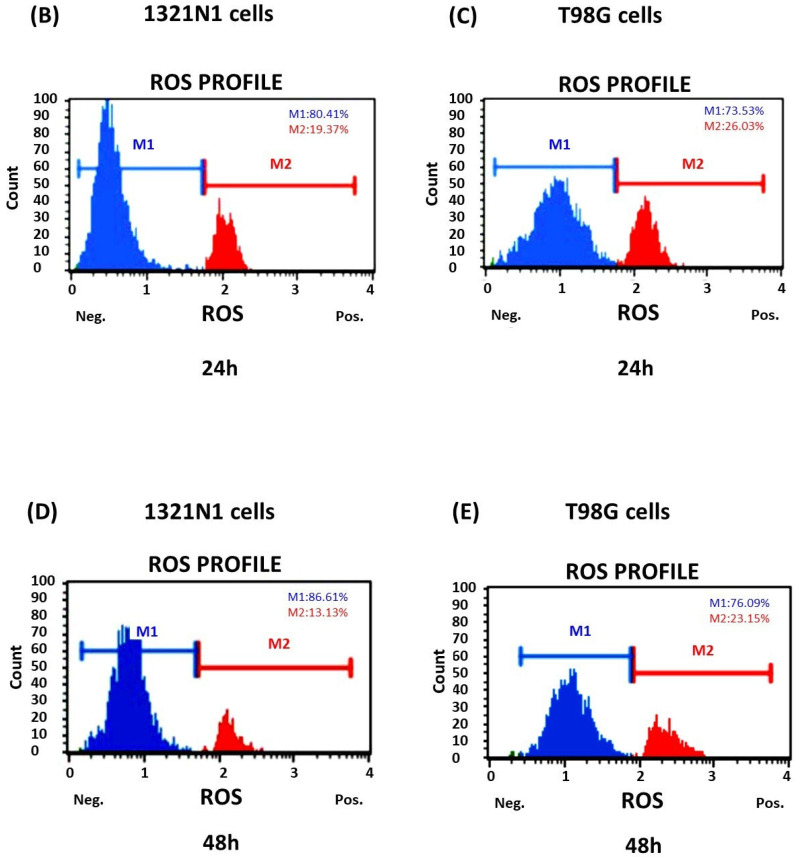
Reactive oxygen species production detected in 1321N1 and T98G cells. The cells were cultured for 24 and 48 h. The percentage of ROS-positive (ROS (+)) and ROS-negative cells (ROS (−)) was assessed. (**A**) The results are expressed as the mean ± SD of three or more independent experiments; an asterisk (*) indicates statistically significant differences (*p* < 0.05) between cell lines among ROS-positive cells; (#) indicates statistically significant differences (*p* < 0.05) between cell lines among ROS-negative cells. (**B**–**E**) Representative results are shown; the blue area (M1) represents ROS-negative cells, and the red area (M2) represents ROS-positive cells.

The flow cytometry analysis showed that the percentage of ROS-positive cells was greater in T98G cells than in 1321N1 cells at both 24 h and 48 h of culture (Figure 4A–E).

## 4. Discussion

In the present study, it has been demonstrated that in T98G glioblastoma cells, the level of sulfane sulfur (Figure 2) as well as the level of ROS production (Figure 4) is higher than in 1321N1 astrocytoma cells.

Both cell lines were cultured under the same standard conditions in high-glucose medium recommended for glioma cells. Glucose metabolism plays an essential role in the development and growth of glioma cells [50]. These conditions, together with oxygen concentration, are not physiological and may also affect ROS production. Glioma cells have a higher rate of aerobic glycolysis than healthy brain tissue, which is more pronounced in glioblastoma multiforme than in low-grade gliomas [51]. The results obtained in vitro should be related to the conditions occurring in tissues with caution.

The rapid growth of brain tumors, such as glioblastoma, often results in oxygen deprivation and the formation of hypoxic zones. Glioblastoma cells can survive and adapt to such a hypoxic environment [52]. Increased levels of reactive oxygen species in GBM promote cancer cell survival and cause drug resistance [7,53]. Wróbel et al. [54] reported that human gliomas with the highest grade of malignancy (III/IV) maintained a high GSH/GSSG ratio, which is important for cancer cell proliferation.

In our work, we showed that in T98G cells, the increased level of ROS is accompanied by an increased level of sulfane sulfur compared to 1321N1 cells (Figure 2). High levels of sulfane sulfur were also found in gliomas with the highest grade of malignancy [54]. In addition, Shiota et al. [55] demonstrated that hydrogen polysulfide (H_2_S_n_) levels were greater in glioblastoma-bearing regions than glioblastoma-free control regions. H_2_S_2_ and H_2_S_3_ exhibit antioxidant activity and anti-carbonyl stress effects by regulating the redox balance in neuronal cells; H_2_S_2_ and H_2_S_3_ promote GSH synthesis, which is dependent on NRF2 [40,56]. NRF2 is associated with cellular progression of brain cancers, such as astrocytomas, multiforme glioblastomas, gliosarcomas, medulloblastomas, and oligodendroglial and ependymal tumors [57]. Tsai et al. reported [58] that in human glioma cell lines, the expression of NRF2 was higher than in normal brain tissue. Furthermore, NRF2 expression in gliomas was positively correlated with WHO grades [58]. NRF2, while cytoprotective, transcriptionally controls iron and heme genes (e.g., ferritin, heme oxygenase 1) and can dampen ferroptotic susceptibility; its sustained activation in glioblastoma multiforme aligns with hypoxic adaptation, HIF-1α/VEGF signaling, and reduced chemosensitivity, whereas NRF2 inhibition restores apoptosis and temozolomide responsiveness [6,8].

Interestingly, our results showed that in T98G glioblastoma cells, the expression of transferrin receptor 1, cystathionine β-synthase, and cysteine dioxygenase 1 (iron-dependent enzymes) is significantly increased compared to 1321N1 astrocytoma cells (Figure 3A,B).

Due to higher rates of proliferation and DNA synthesis, cancer cells have a greater demand for iron than normal cells [59]. Among brain tumors, astrocytomas clearly express TfR1, with glioblastoma multiforme showing the highest expression [12]. TfR1 in gliomas increases iron accumulation and promotes tumor progression by increasing the proliferation rates and glutamate production [12]. Cancer cells typically up-regulate TfR1 and down-regulate ferroportin to retain iron, creating a labile iron pool that fuels proliferation yet predisposes to ferroptosis upon antioxidant collapse [59]. Ferritin-based nanovectors that engage TfR1 can traverse the blood–brain barrier and, via intranasal routes, concentrate cytotoxic payloads within high-TfR1 gliomas, improving survival in vivo [60]. Innovative therapeutic strategies (such as iron chelation therapies, ferroptosis induction, to nanoparticle-based drug delivery) targeting iron dysregulation offer hope in the treatment of glioblastoma multiforme (GBM) [14].

Zhong et al. [61] reported that CBS activity is up-regulated by oxidative stress in primary rat cortical neurons. CBS can be S-glutathionylated (Cys346-SH→Cys346-SOH→Cys346-SSG) under oxidative stress, resulting in increased CBS activity and subsequent H_2_S production [10,62].

Cystathionine β-synthase is required for iron homeostasis [63]. Zhou et al. [63] demonstrated that CBS knockout mice exhibited anemia, a significant increase in iron content in the serum, liver, spleen, and heart, and liver damage. CBS deficiency markedly reduced the expression of TfR1 and NRF2 and GSH content in the liver [63].

Endogenous H_2_S production by CBS supports tumor growth by maintaining mitochondrial respiration and ATP synthesis, stimulating cell proliferation and survival and redox regulation (promotes antioxidant production by enhancing NRF2 activation and increasing glutathione production), and modulating protein activity via protein S-sulfhydration [64]. It is therefore possible that high CBS expression (Figure 3A,B) as well as high levels of sulfane sulfur (Figure 2) in T98G glioblastoma cells allow them to maintain a high antioxidant capacity.

CBS catalyzes the condensation of homocysteine with serine or cysteine to form cystathionine [34,65]. Elevated cystathionine levels were observed in vivo in IDH-mutated 1p/19q co-deleted gliomas compared with their non-co-deleted counterparts and normal brain tissue [66]. Our previous studies [67] showed that cystathionine promotes the proliferation of human astrocytoma U373 cells in a time dependent-manner, which was associated with increased intracellular L-cysteine and L-cystine levels and the GSH/GSSG ratio.

Due to the high expression of CBS in T98G cells, it appears that CBS is an important enzyme for cysteine synthesis in these cells. Cancer cells with higher ROS levels activate the intracellular antioxidant systems to maintain redox balance [68]. CBS may promote cancer cell survival by increasing their internal antioxidant capacity [64]. CBS is responsible for regulating the synthesis of L-cysteine, which is the precursor of GSH, a major intracellular antioxidant [68]. The level of GSH in glioblastoma cells is higher than in normal cells [69]. In glioblastoma cells with high expression of apolipoprotein C1, the expression of CBS can be increased, which can stimulate the transsulfuration pathway, increase GSH synthesis, and induce ferroptosis resistance [70]. Additionally, overexpression of γ-glutamyl transferase as well as xCT (SLC7A11) transporter in glioblastoma can maintain GSH homeostasis and protect tumor cells from oxidative stress [69]. Methionine is taken up and metabolized at higher rates by glioma cells; L-cysteine catabolic pathways are up-regulated in glioblastoma [71]. In vitro studies [72] showed that cysteine and methionine deprivation can decrease the levels of GSH, hypotaurine, and taurine, and induce ferroptosis of glioma cells. The growth of glioma cells was also inhibited when GSH synthesis was blocked by buthionine sulfoximine (an inhibitor of γ-glutamylcysteine ligase required for glutathione synthesis) [73,74].

In this study, we demonstrated the higher expression of cysteine dioxygenase 1 in T98G glioblastoma cells compared to 1321N1 astrocytoma cells (Figure 3A,B).

CDO1, a main enzyme in the oxidative pathway of L-cysteine, is involved in the formation of cysteine sulfinic acid, which consequently leads to hypotaurine production (a sulfur-containing amino acid derivative, which exhibits antioxidant properties) [30,75].

Studies of Prabhu et al. [76] conducted on patient-derived glioma specimens showed the accumulation of cysteine sulfinic acid (a product of reaction catalyzed by CDO1) in glioblastoma; CSA was identified as one of the main metabolites differentiating glioblastoma from low-grade glioma. High CSA levels were correlated with high CDO1 expression in grade 4 glioma compared with grade 2 glioma; both GSH and hypotaurine levels were also higher in glioblastoma. Interestingly, the inhibition of CDO1 attenuates glioblastoma growth in vivo [76].

Gao et al. [77] demonstrated that tissue hypotaurine levels strongly and positively correlated with glioma grade; it was associated with increased expression of X_C_-glutamate-cystine antiporter in glioma tissues. In vitro studies showed that hypotaurine increased the proliferative and migratory capacity of U251 and U87 glioma cells; hypotaurine activates hypoxia signaling in glioma cells through the competitive inhibition of prolyl hydroxylase domain-2, leading to HIF α stabilization [77]. Moreover, taurine supplementation led to decreased intracellular hypotaurine levels and concomitant inhibition of U251 cell growth [77].

Recent studies [78] have shown that hypotaurine promotes glioma U251 cell invasion by epigenetically regulating Wnt5a expression (Wnt5a, a glycoprotein that functions as a tumor suppressor or promoter, depending on the cancer type). In U251 cells, hypotaurine affects the reduction in Wnt5a expression and the methylation of the Wnt5a promoter via the possible inhibition of demethylases activity [78].

Cysteine catabolism into taurine and sulfate—or into H_2_S and its oxidation products—maintains sulfur homeostasis but also generates toxic intermediates. Sulfite accumulation can inhibit mitochondrial dehydrogenases and amplify ROS, while aberrant S-sulfonate formation drives neurotoxicity [28].

Taurine, a key osmolyte and antioxidant, arises via cysteine dioxygenase and cysteine sulfinic acid decarboxylase activity in neurons and astrocytes but is largely imported by transformed astrocytomas like 1321N1 cells [79]. Under hypertonic or oxidative stress, normal glia ramp up both the synthesis and uptake of taurine, whereas glioma cells depend predominantly on transport [79]. Cao et al. [80] reported that the taurine transporter SLC6A6 is associated with tumor progression; high SLC6A6 expression was correlated with poor overall survival and progression-free survival in glioblastoma, low-grade glioma, as well as in gastric, liver, and pancreatic cancers. Therefore, inhibition of the taurine transporter could deprive tumors of this protective osmolyte and antioxidant, exacerbating oxidative stress.

In 1321N1 and T98G cells, we demonstrated the expression of CTH, MPST, TST, and p53 protein (Figure 3A,C); however, we did not observe changes in the expression of these proteins between the two cell lines.

Cystathionine γ-lyase converts cystathionine to cysteine, which is also a substrate for GSH synthesis [28]. CTH was specifically up-regulated in IDH1-mutant astrocytomas to sustain de novo GSH production under cysteine restriction, and CTH inhibition with brain-penetrant propargylglycine impaired tumor growth in vivo [16].

Saurty-Seerunghen et al. [18] found that enhanced ROS production and 3-mercaptopyruvate sulfurtransferase activity are required for glioblastoma cell motility. MPST protects protein cysteine residues from dismal hyperoxidation; in vivo, MPST knockout increases mice survival [18].

Mitochondrial thiosulfate sulfurtransferase also exerts antioxidant properties [81]. In a murine model (Tst^−/−^ mice), TST deficiency in the brain perturbs ROS/RSS (notably polysulfides) generation, remodels oxidative phosphorylation, and deranges NRF2–KEAP1 signaling, rendering neural tissue more susceptible to oxidative distress [17].

p53 protein plays a key role in cell oxidative stress, DNA repair, and iron metabolism [63]. p53 protein contains cysteine residues located on its surface which function as redox sensors [82,83,84]. Excess iron can transform the genome and epigenome by disrupting the p53-dependent DNA repair pathway and increasing DNA hypomethylation [59]. p53 can suppress ferroptosis through transcription-dependent and transcription-independent mechanisms, suggesting bidirectional and context-dependent control of ferroptosis and oxidative stress by p53 [59]. The TP53 gene is mutated in both 1321N1 and T98G cells. Mutant p53 sustains an increase in intracellular ROS, affecting the cellular redox balance and promoting cancer survival [84].

Our findings on iron addiction and redox balance occur alongside broader shifts in growth-factor signaling networks. A loss of PTEN (a tumor suppressor gene)—and the resulting chronic elevation of phosphatidylinositol (3,4,5)-trisphosphate—selectively desensitizes IGF-1 and insulin receptor pathways, while leaving EGF/PDGF responses intact through down-regulation of IRS1/2 and receptor expression [85]. In PTEN-mutated cells, this adaptive feedback could blunt upstream survival cues from IGF-1, rendering cells more reliant on alternative trophic inputs (e.g., transferrin-mediated iron uptake) and potentially sensitizing them to combined PI3K/Akt and iron-targeted therapies.

Together, these data position oxidative stress as well as sulfur and iron biochemistry (in particular, TfR1, CBS, CDO1, CTH, MPST, TST, p53) as interconnected determinants of glioma behavior and as rational foundations for studies probing how selective manipulation of ROS, sulfane sulfur signaling, and iron transport can be leveraged to constrain invasion, re-sensitize tumors, and improve outcomes.

## 5. Conclusions

The present study revealed that in T98G glioblastoma cells, the levels of sulfane sulfur and ROS production are higher than in 1321N1 astrocytoma cells. Western blot analysis showed the up-regulation of cystathionine β-synthase, cysteine dioxygenase 1, and the transferrin receptor 1 in T98G glioblastoma cells compared to 1321N1 astrocytoma cells, while the expression of thiosulfate sulfurtransferase, cystathionine γ-lyase, 3-mercaptopyruvate sulfurtransferase, and p53 protein was comparable in both cell lines.

High production of ROS in T98G cells is associated with higher levels of sulfane sulfur, which could facilitate glioblastoma cells in maintaining a high antioxidant capacity. High CBS and CDO1 expression, as well as high levels of sulfane sulfur in T98G glioblastoma cells, suggests that sulfur metabolism is a critical determinant of tumor redox balance. It appears that CBS and CDO1 may significantly increase the antioxidant potential of T98G cells. CBS may affect the increase in L-cysteine levels and thus GSH production in glioblastoma cells. On the other hand, increased CDO1 expression may influence the conversion of L-cysteine to hypotaurine and taurine, which may also play an important antioxidant role in these cells. Therefore, it is possible that modulation of the activity of both CBS and CDO1 may affect the antioxidant potential and survival of T98G glioblastoma cells, which we intend to verify in further studies.

The differential expression of CBS, CDO1, and TfR1 underscores a metabolic vulnerability in high-grade gliomas. The overall findings indicate that oxidative stress, together with sulfur and iron biochemistry, functions as an interacting determinant regulating glioma phenotype, providing a basis supporting research targeting reactive oxygen species production, sulfane sulfur, signal transduction, and iron transport that can be leveraged to constrain invasion, re-sensitize tumors, and improve outcomes.

## Figures and Tables

**Table 1:** Biological and genetic characteristics of 1321N1 and T98G glioma cell lines (based on https://www.culturecollections.org.uk/ecacc; https://www.cellosaurus.org; accessed on 1 September 2025) [46,47].

Characteristic	1321N1 Cells	T98G Cells
**Tissue of Origin**	Brain	Brain
**Cell Line Description**	1321N1: originally thought to derive from U-118 MG; U-118 MG itself was derived from U-138 MG (astrocytoma).	T98G cell line was derived from glioblastoma multiforme tumor from a 61-year-old Caucasian male
**Disease**	Astrocytoma	Glioblastoma
**Growth Mode**	Adherent	Adherent
**DNA Profile** **(STR Profile)**	Amelogenin: X CSF1PO: 11,12 D5S818: 11 D7S820: 9 D13S317: 9,11 D16S539: 12,13 TH01: 6 TPOX: 8 vWA: 18	Amelogenin: X,Y CSF1PO: 10,12 D5S818: 10,12 D7S820: 9,10 D13S317: 13 D16S539: 13 TH01: 7,9.3 TPOX: 8 vWA: 17,20
**Karyotype**	Not specified	Hyperpentaploid: modal no. 128–132
**Mutation**	TP53, PTEN	TP53, PTEN

## Data Availability

The original contributions presented in this study are included in the article/Supplementary Materials. Further inquiries can be directed to the corresponding author.

## Supplementary Materials

The following supporting information can be downloaded at https://www.mdpi.com/article/10.3390/biom15101478/s1, Figure S1: Original images of Western blot results.
