# Supplementary material for: Oxidative Stress and Iron Addiction: A Comparative Study of 1321N1 Astrocytoma and T98G Glioblastoma Cells with Differential Expression of L-Cysteine-Metabolizing Enzymes
Source: Biomolecules. 2025 Oct 20;15(10):1478. doi: 10.3390/biom15101478 (PMC12563529; doi:10.3390/biom15101478)
Supplement: Supplementary file 1 [file biomolecules-15-01478-s001.zip › biomolecules-3901113-supplementary.pdf]

WESTERN BLOT

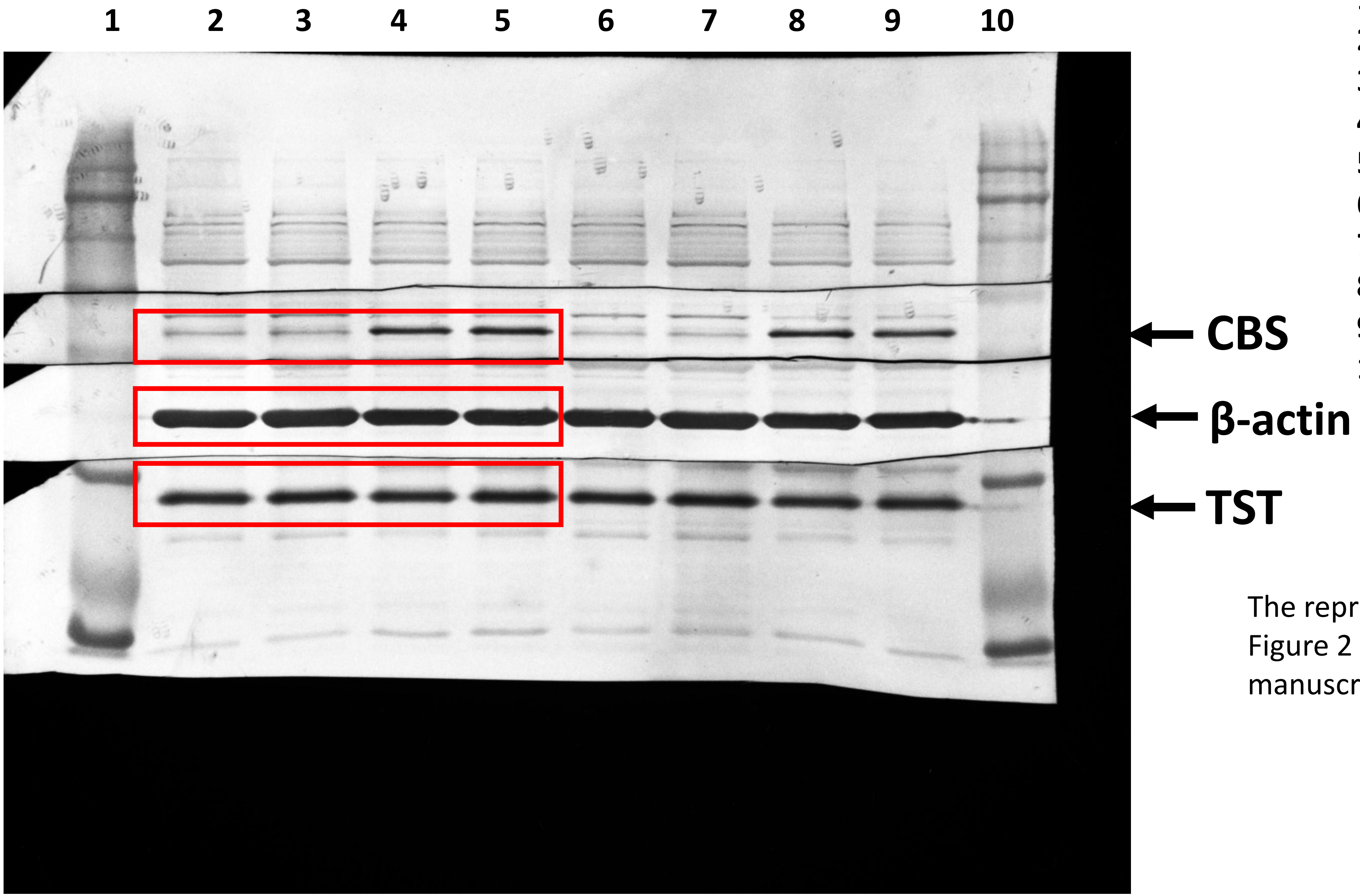

1.Molecular weight standard
2. 1321N1, Control, 24h
3. 1321N1, Control, 48h
4. T98G, Control, 24h
5. T98G, Control, 48h
6. 1321N1, Control, 24h
7. 1321N1, Control, 48h
8. T98G, Control, 24h
9. T98G, Control, 48h
10. Molecular weight standard

The representative results shown in Figure 2 in the main text of the manuscript are marked in red.

WESTERN BLOT

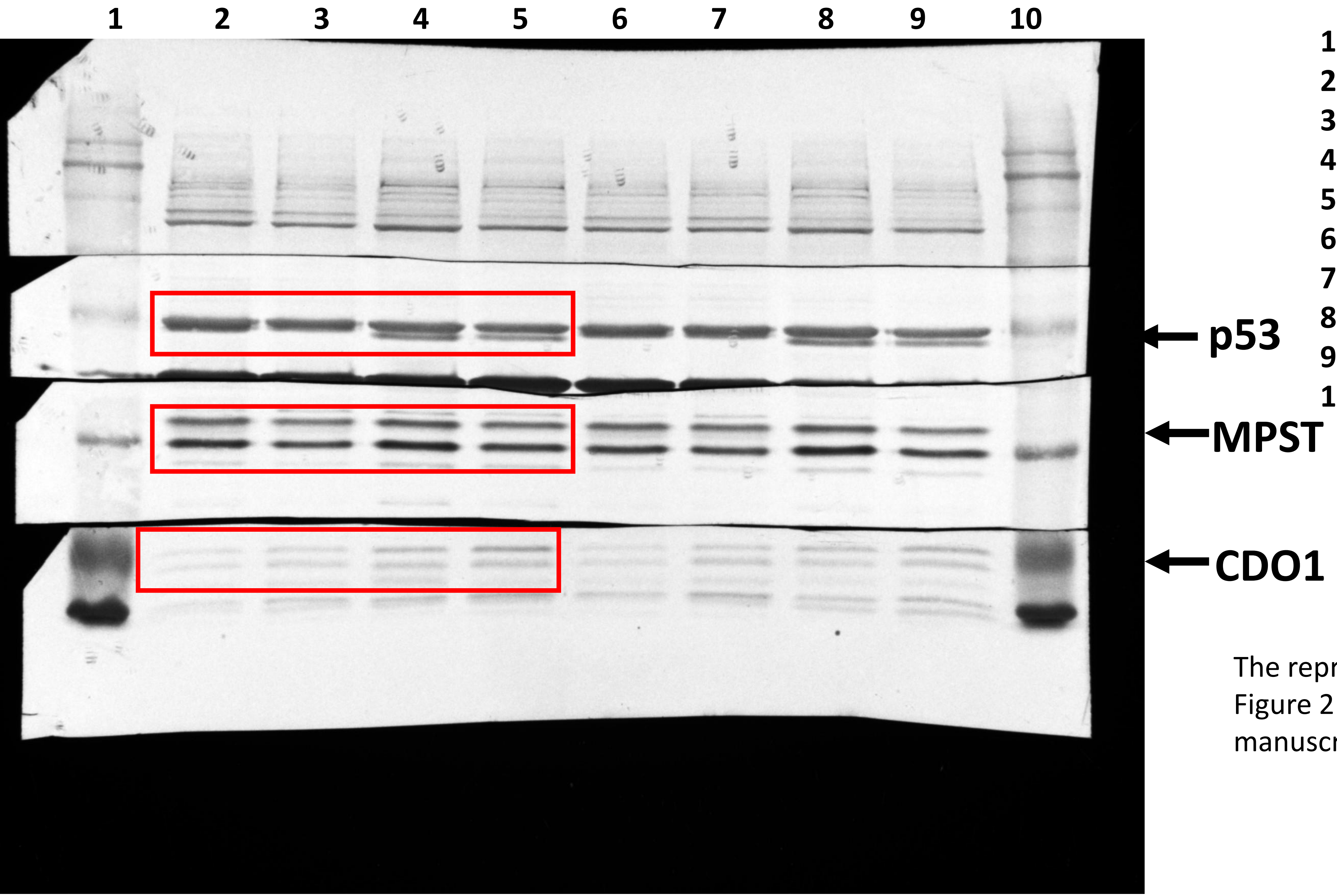

1.Molecular weight standard
2. 1321N1, Control, 24h
3. 1321N1, Control, 48h
4. T98G, Control, 24h
5. T98G, Control, 48h
6. 1321N1, Control, 24h
7. 1321N1, Control, 48h
8. T98G, Control, 24h
9. T98G, Control, 48h
10. Molecular weight standard

The representative results shown in Figure 2 in the main text of the manuscript are marked in red.

WESTERN BLOT

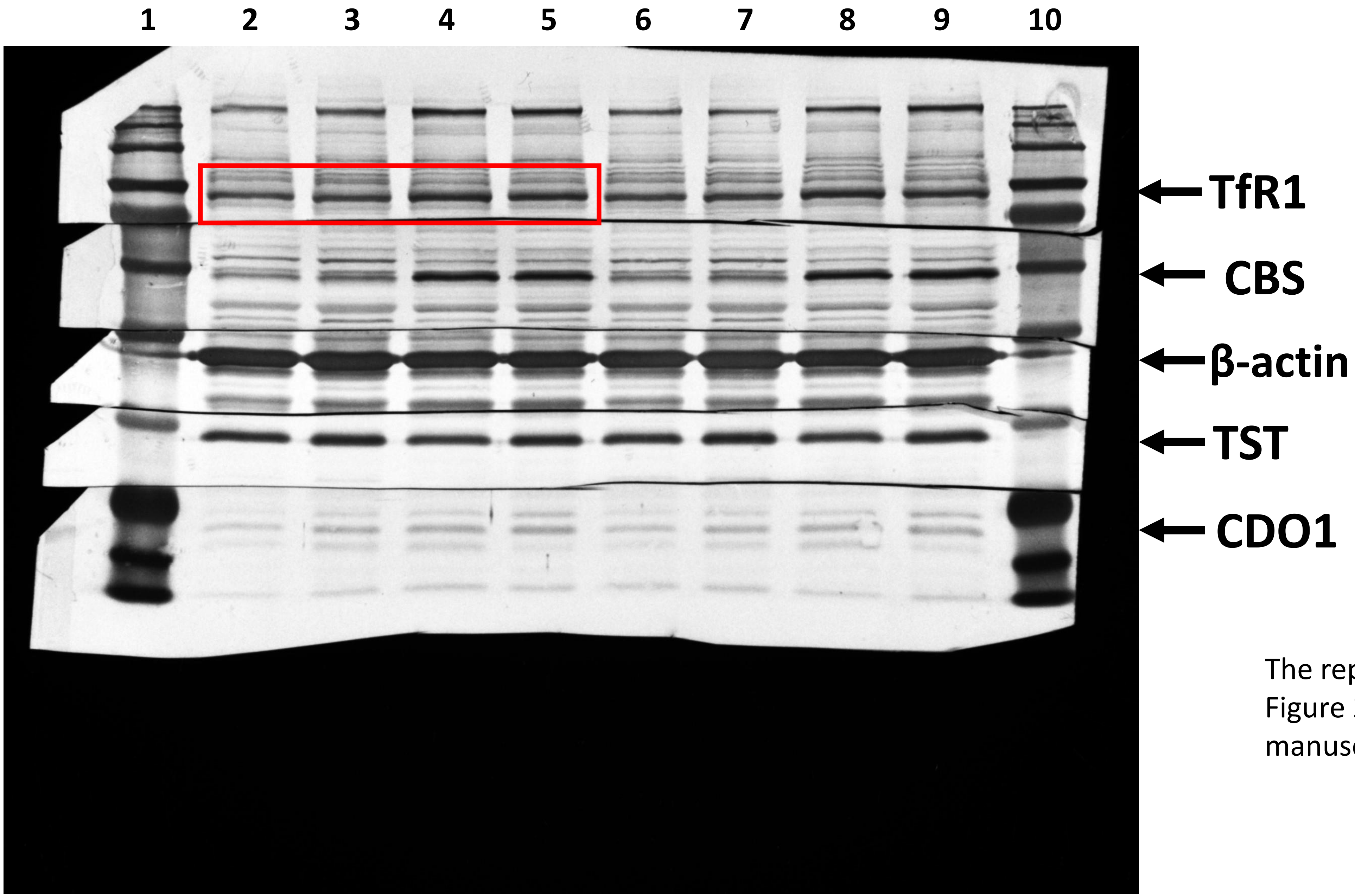

1.Molecular weight standard
2. 1321N1, Control, 24h
3. 1321N1, Control, 48h
4. T98G, Control, 24h
5. T98G, Control, 48h
6. 1321N1, Control, 24h
7. 1321N1, Control, 48h
8. T98G, Control, 24h
9. T98G, Control, 48h
10. Molecular weight standard

The representative results shown in Figure 2 in the main text of the manuscript are marked in red.

WESTERN BLOT

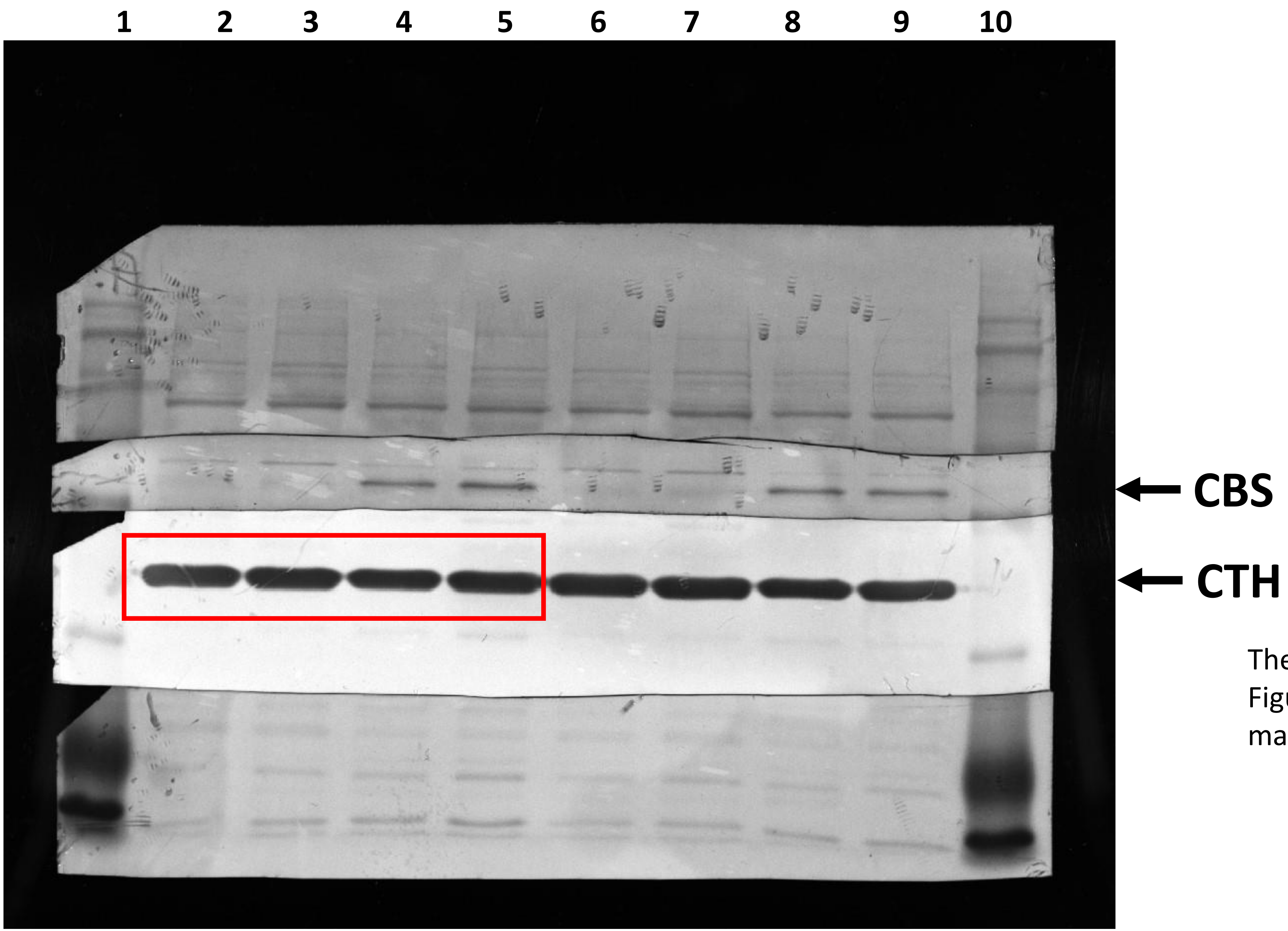

1. Molecular weight standard
2. 1321N1, Control, 24h
3. 1321N1, Control, 48h
4. T98G, Control, 24h
5. T98G, Control, 48h
6. 1321N1, Control, 24h
7. 1321N1, Control, 48h
8. T98G, Control, 24h
9. T98G, Control, 48h
10. Molecular weight standard

The representative results shown in Figure 2 in the main text of the manuscript are marked in red.

WESTERN BLOT

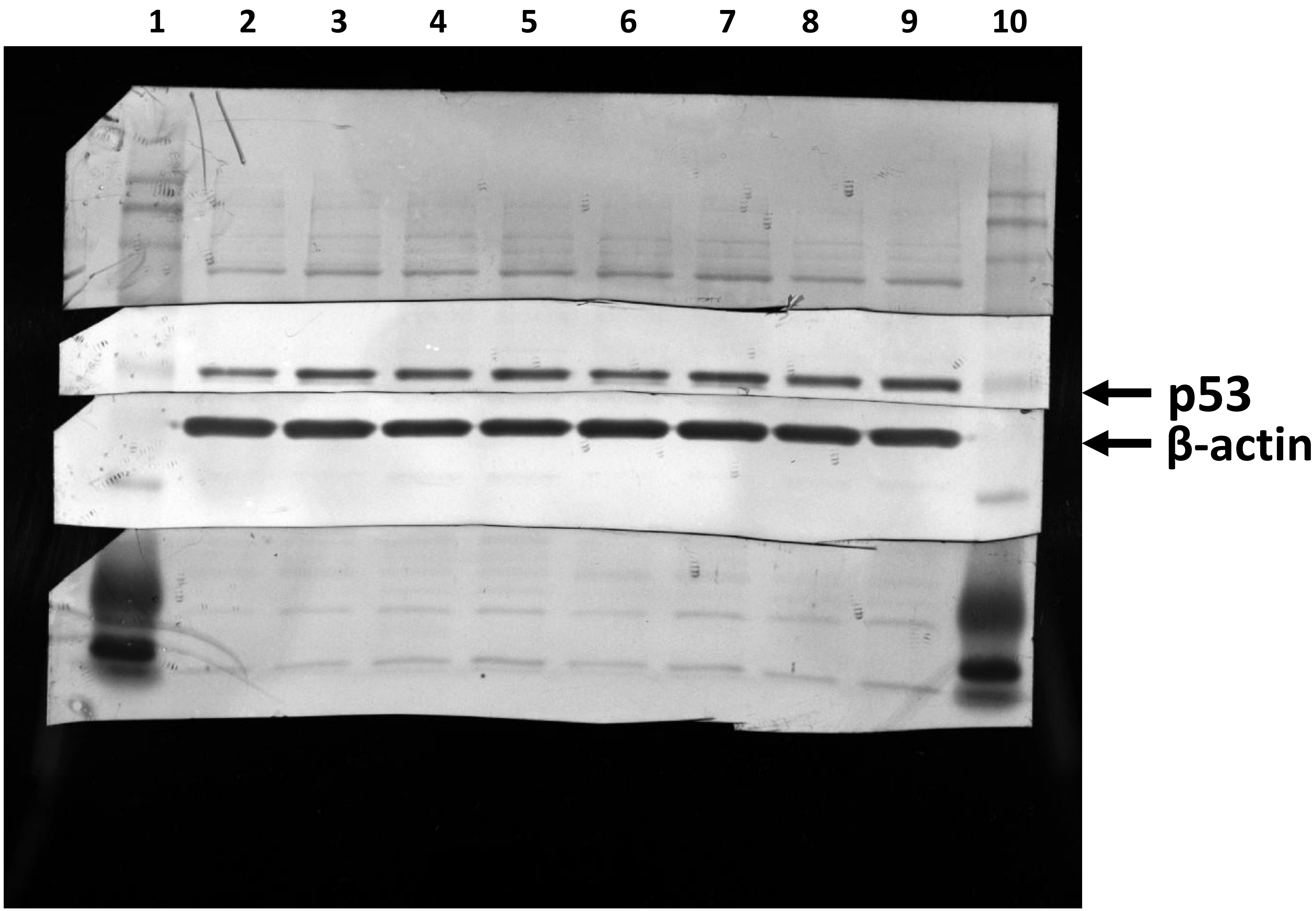

1.Molecular weight standard
2. 1321N1, Control, 24h
3. 1321N1, Control, 48h
4. T98G, Control, 24h
5. T98G, Control, 48h
6. 1321N1, Control, 24h
7. 1321N1, Control, 48h
8. T98G, Control, 24h
9. T98G, Control, 48h
10. Molecular weight standard

WESTERN BLOT

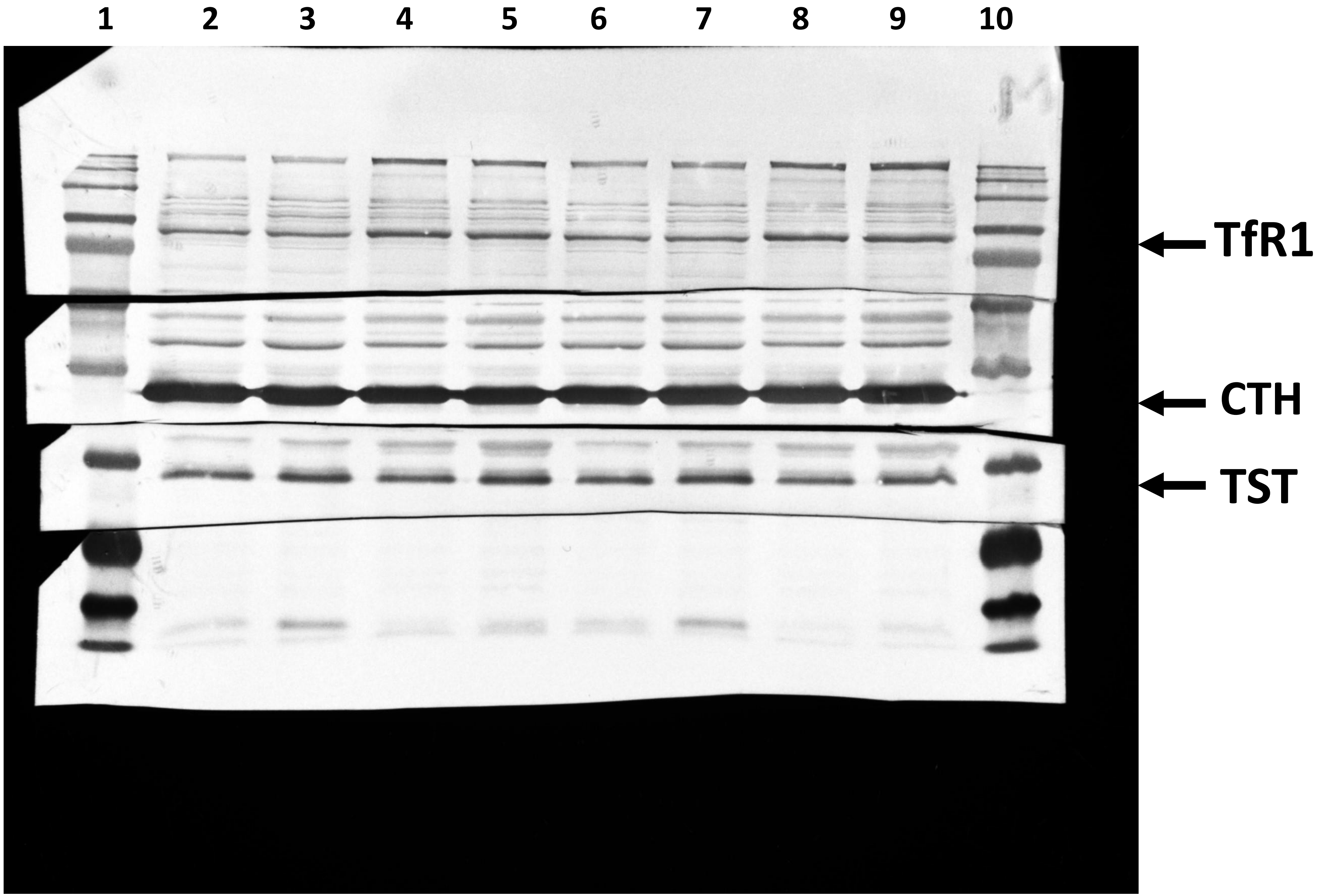

1. Molecular weight standard
2. 1321N1, Control, 24h
3. 1321N1, Control, 48h
4. T98G, Control, 24h
5. T98G, Control, 48h
6. 1321N1, Control, 24h
7. 1321N1, Control, 48h
8. T98G, Control, 24h
9. T98G, Control, 48h
10. Molecular weight standard

WESTERN BLOT

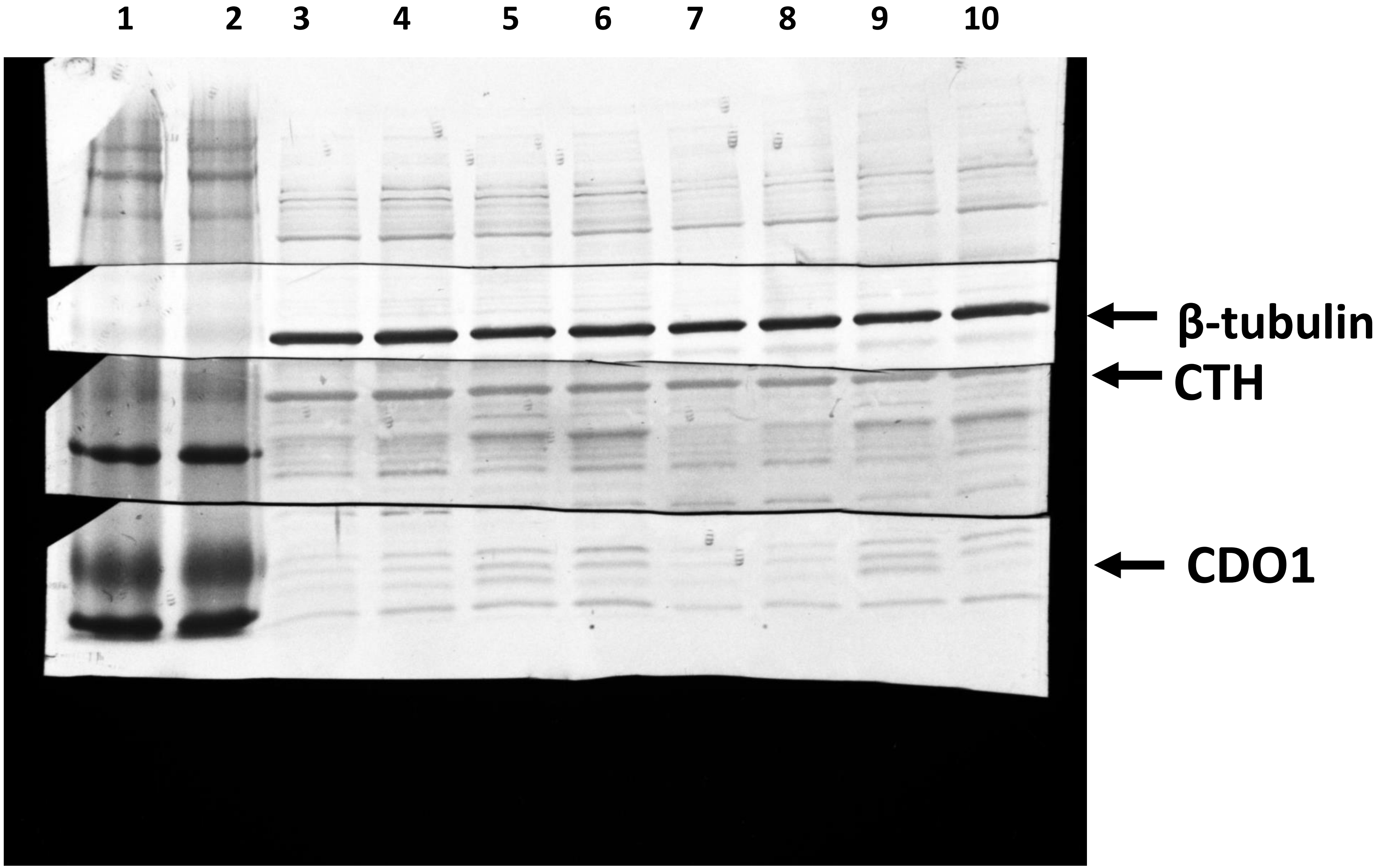

1.Molecular weight standard
2. Molecular weight standard
3. 1321N1, Control, 24h
4. 1321N1, Control, 48h
5. T98G, Control, 24h
6.T98G, Control, 48h
7.1321N1, Control, 24h
8.1321N1, Control, 48h
9. T98G, Control, 24h
10. T98G, Control, 48h

WESTERN BLOT

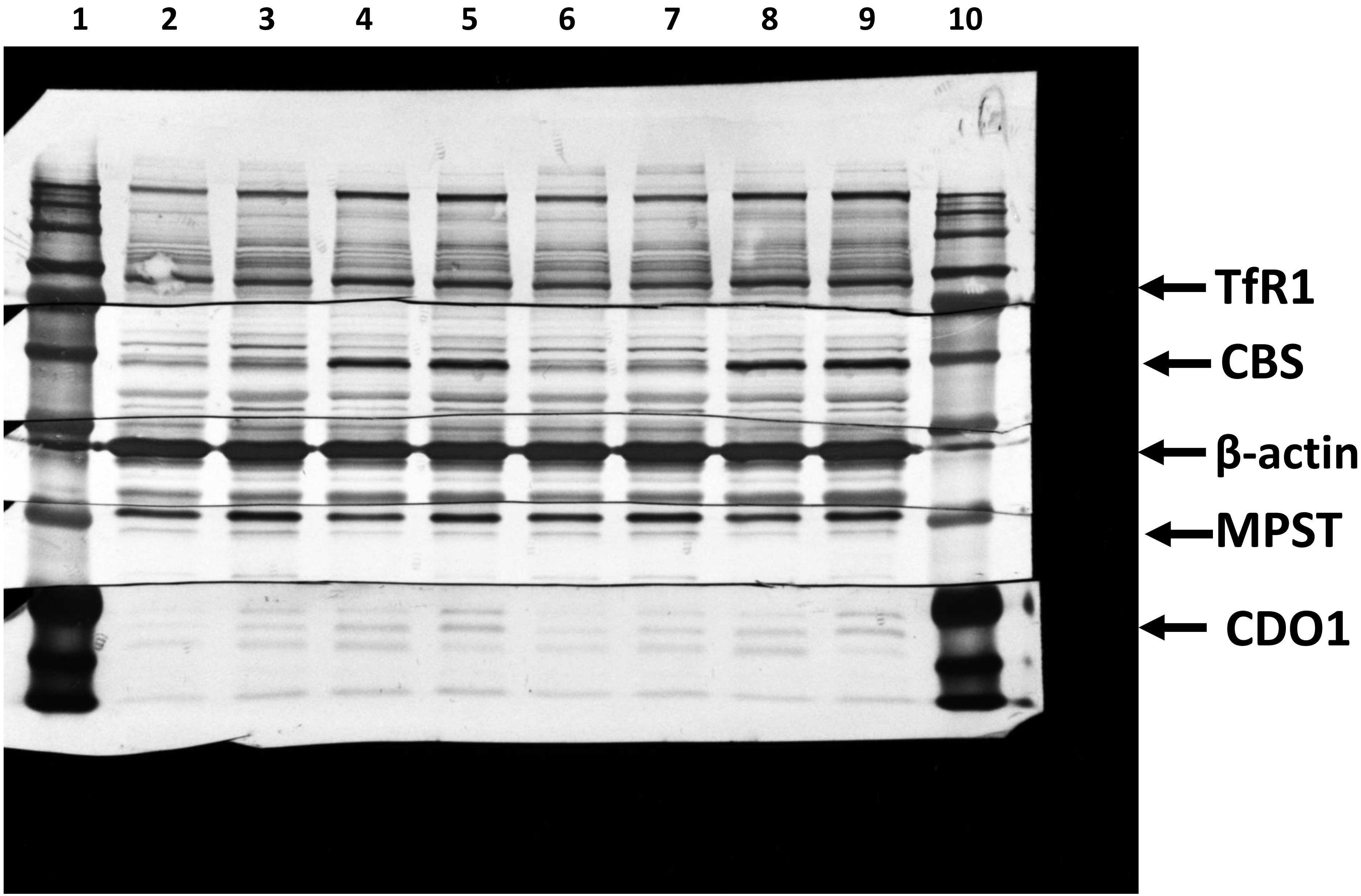

1. Molecular weight standard
2. 1321N1, Control, 24h
3. 1321N1, Control, 48h
4. T98G, Control, 24h
5. T98G, Control, 48h
6. 1321N1, Control, 24h
7. 1321N1, Control, 48h
8. T98G, Control, 24h
9. T98G, Control, 48h
10. Molecular weight standard

WESTERN BLOT

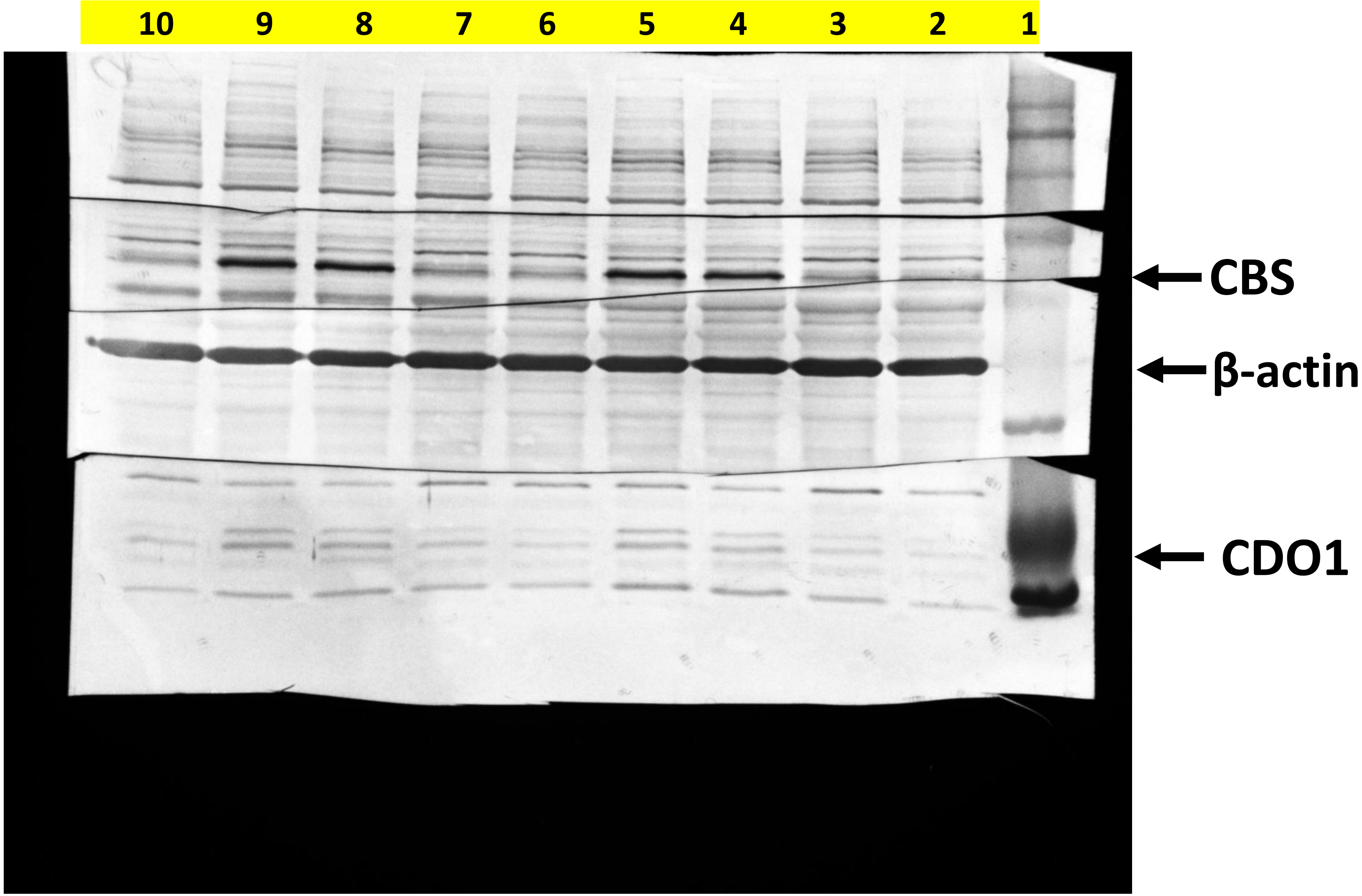

1. Molecular weight standard
2. 1321N1, Control, 24h
3. 1321N1, Control, 48h
4. T98G, Control, 24h
5. T98G, Control, 48h
6. 1321N1, Control, 24h
7. 1321N1, Control, 48h
8. T98G, Control, 24h
9. T98G, Control, 48h
10. 1321N1, Control

WESTERN BLOT

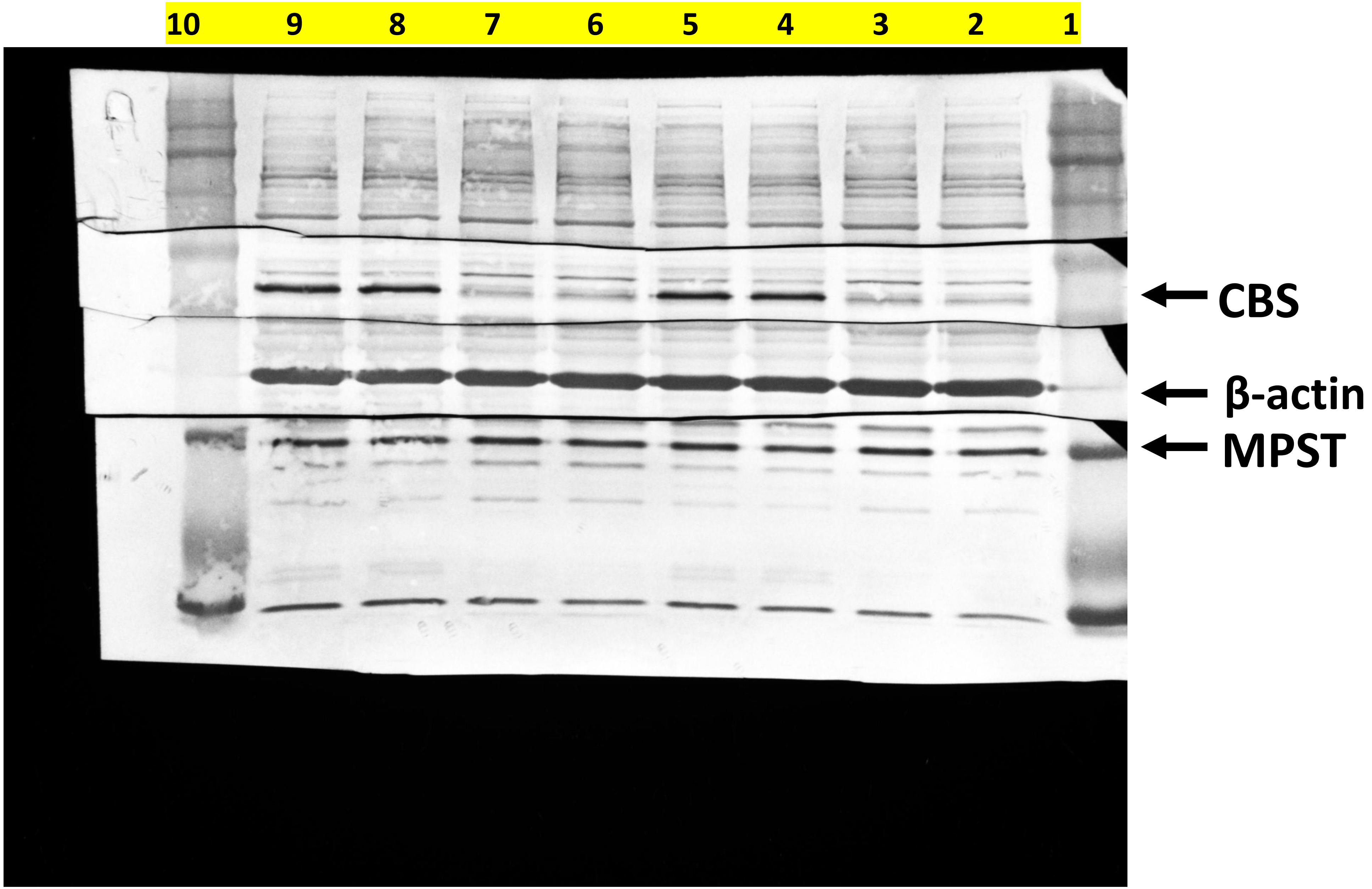

1.Molecular weight standard
2. 1321N1, Control, 24h
3. 1321N1, Control, 48h
4. T98G, Control, 24h
5. T98G, Control, 48h
6. 1321N1, Control, 24h
7. 1321N1, Control, 48h
8. T98G, Control, 24h
9. T98G, Control, 48h
10. Molecular weight standard

WESTERN BLOT

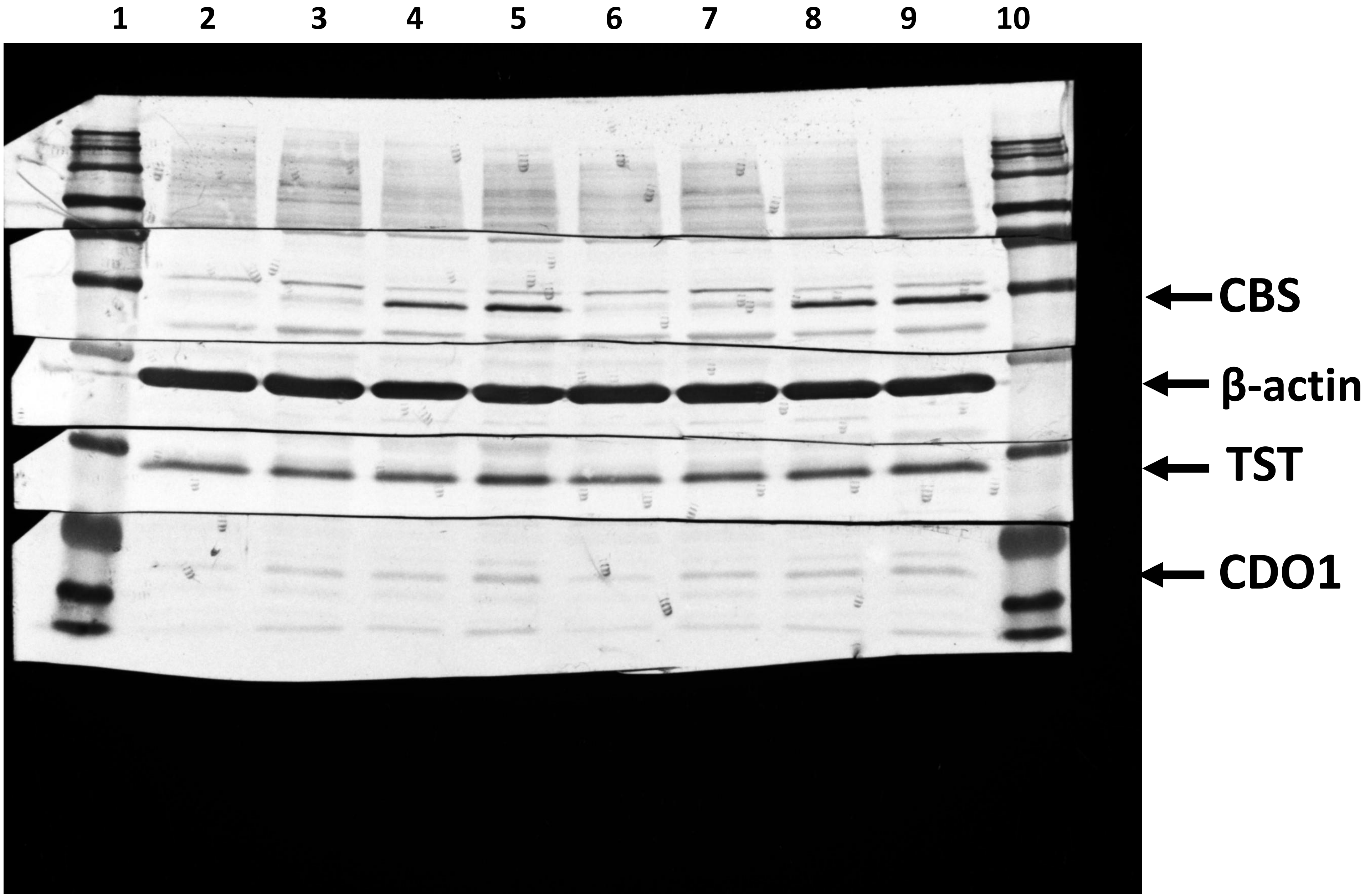

1.Molecular weight standard
2. 1321N1, Control, 24h
3. 1321N1, Control, 48h
4. T98G, Control, 24h
5. T98G, Control, 48h
6. 1321N1, Control, 24h
7. 1321N1, Control, 48h
8. T98G, Control, 24h
9. T98G, Control, 48h
10. Molecular weight standard

WESTERN BLOT

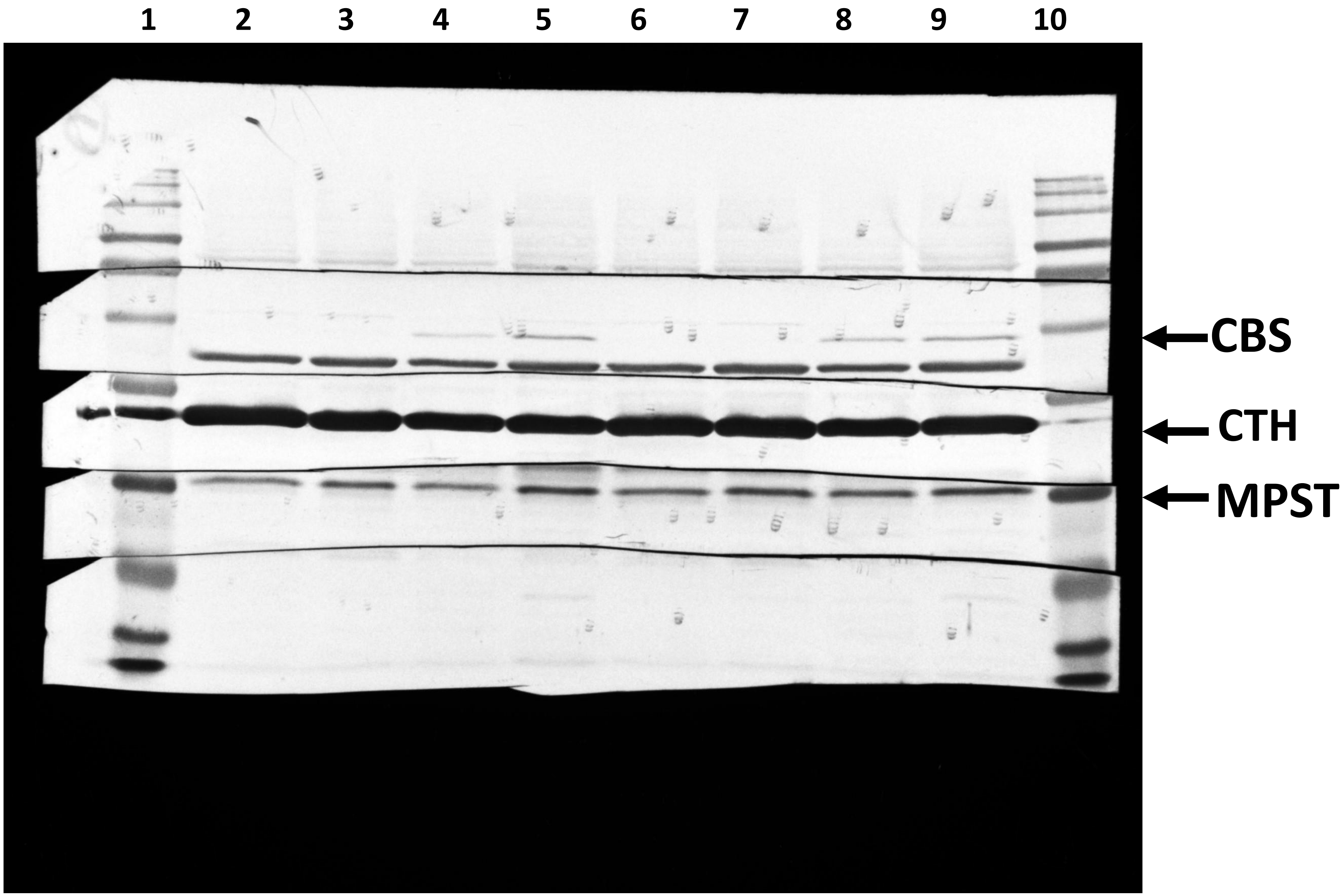

1. Molecular weight standard
2. 1321N1, Control, 24h
3. 1321N1, Control, 48h
4. T98G, Control, 24h
5. T98G, Control, 48h
6. 1321N1, Control, 24h
7. 1321N1, Control, 48h
8. T98G, Control, 24h
9. T98G, Control, 48h
10. Molecular weight standard

WESTERN BLOT

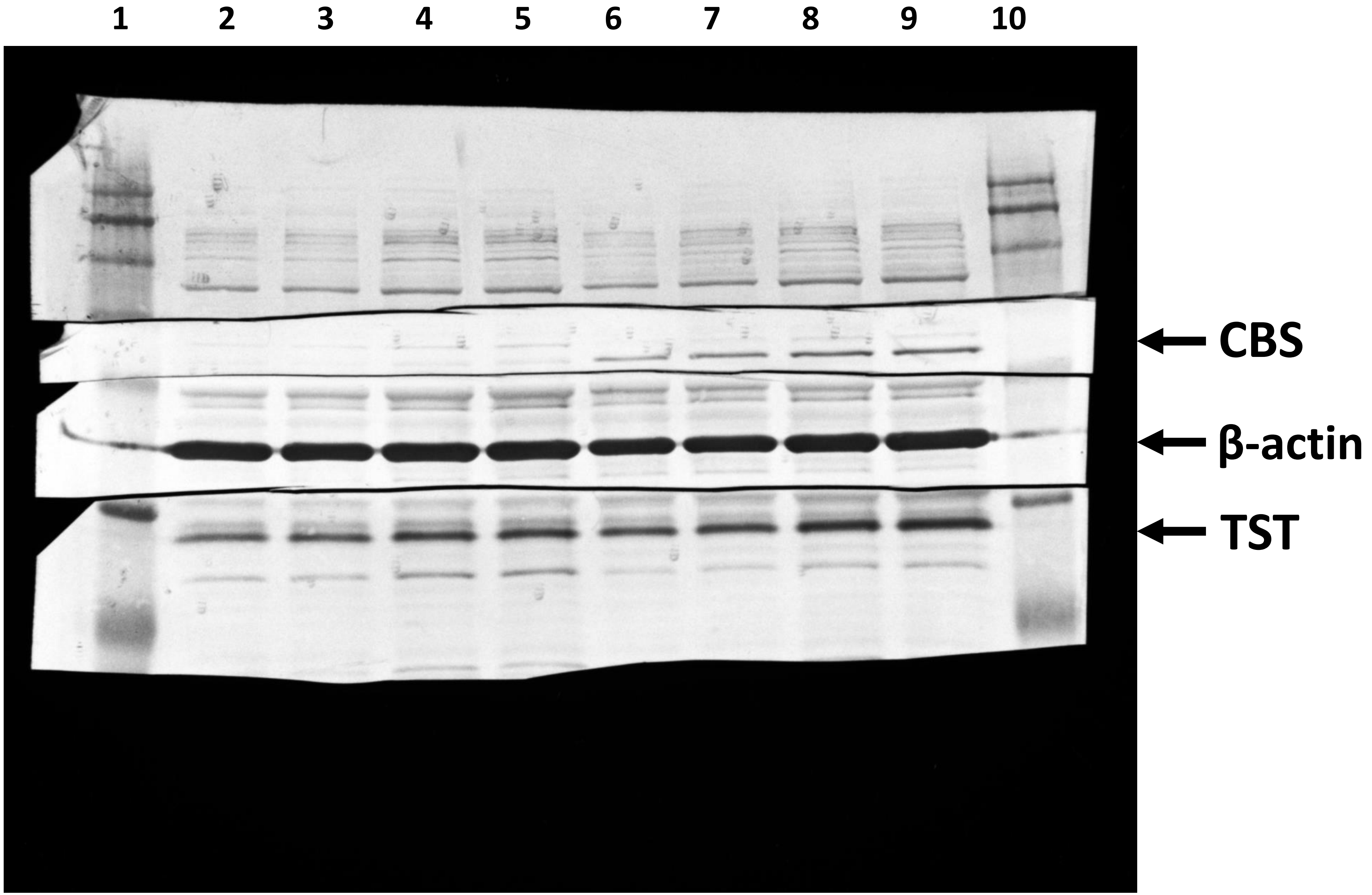

1.Molecular weight standard
2. 1321N1, Control, 24h
3. 1321N1, Control, 24h
4. 1321N1, Control, 48h
5 1321N1, Control, 48h
6. T98G, Control, 24h
7. T98G, Control, 24h
8. T98G, Control, 48h
9. T98G, Control, 48h
10. Molecular weight standard

WESTERN BLOT

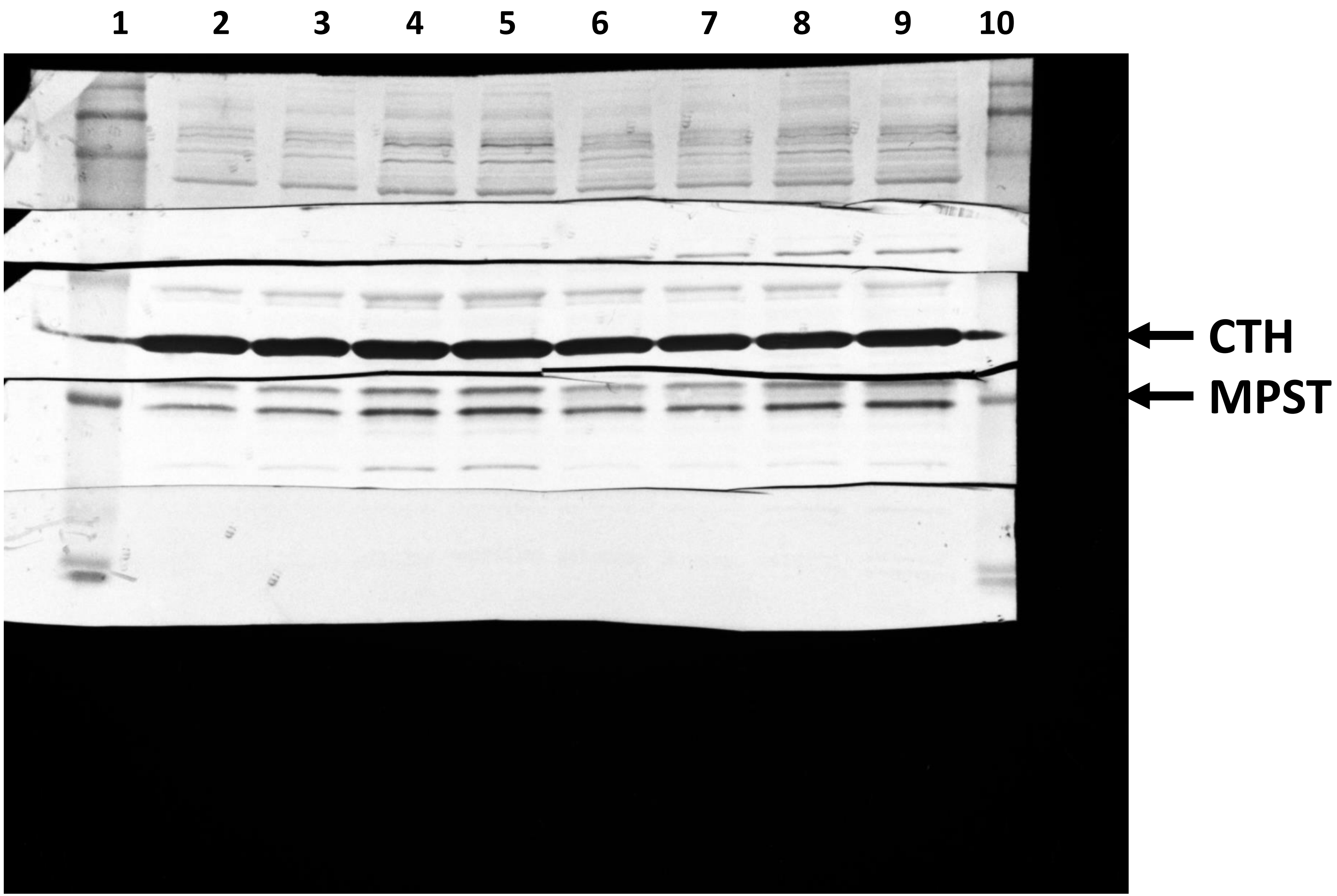

1.Molecular weight standard
2. 1321N1, Control, 24h
3. 1321N1, Control, 24h
4. 1321N1, Control, 48h
5 1321N1, Control, 48h
6. T98G, Control, 24h
7. T98G, Control, 24h
8. T98G, Control, 48h
9. T98G, Control, 48h
10. Molecular weight standard
